# Bulk brain tissue cell-type deconvolution with bias correction for single-nuclei RNA sequencing data using DeTREM

**DOI:** 10.1186/s12859-023-05476-w

**Published:** 2023-09-19

**Authors:** Nicholas K. O’Neill, Thor D. Stein, Junming Hu, Habbiburr Rehman, Joshua D. Campbell, Masanao Yajima, Xiaoling Zhang, Lindsay A. Farrer

**Affiliations:** 1 Bioinformatics Program, Boston University, Boston, MA USA; 2 Department of Mathematics and Statistics, Boston University, Boston, MA USA; 3 Department of Medicine (Biomedical Genetics), Boston University, Chobanian & Avedisian School of Medicine, Boston, MA USA; 4 Department of Medicine (Computational Biomedicine), Boston University Chobanian & Avedisian School of Medicine, Boston, MA USA; 5 Department of Pathology and Laboratory Medicine, Boston University Chobanian & Avedisian School of Medicine, Boston, MA USA; 6 Department of Neurology, Boston University Chobanian & Avedisian School of Medicine, Boston, MA USA; 7 Department of Ophthalmology, Boston University Chobanian & Avedisian School of Medicine, Boston, MA USA; 8 Department of Biostatistics, Boston University School of Public Health, Boston, MA USA; 9 Department of Epidemiology, Boston University School of Public Health, Boston, MA USA; 10 Veterans Administration Medical Center, Bedford, MA USA

**Keywords:** Deconvolution, Single-nuclei RNA sequencing, Brain cell-types, MuSiC

## Abstract

**Background:**

Quantifying cell-type abundance in bulk tissue RNA-sequencing enables researchers to better understand complex systems. Newer deconvolution methodologies, such as MuSiC, use cell-type signatures derived from single-cell RNA-sequencing (scRNA-seq) data to make these calculations. Single-nuclei RNA-sequencing (snRNA-seq) reference data can be used instead of scRNA-seq data for tissues such as human brain where single-cell data are difficult to obtain, but accuracy suffers due to sequencing differences between the technologies.

**Results:**

We propose a modification to MuSiC entitled ‘DeTREM’ which compensates for sequencing differences between the cell-type signature and bulk RNA-seq datasets in order to better predict cell-type fractions. We show DeTREM to be more accurate than MuSiC in simulated and real human brain bulk RNA-sequencing datasets with various cell-type abundance estimates. We also compare DeTREM to SCDC and CIBERSORTx, two recent deconvolution methods that use scRNA-seq cell-type signatures. We find that they perform well in simulated data but produce less accurate results than DeTREM when used to deconvolute human brain data.

**Conclusion:**

DeTREM improves the deconvolution accuracy of MuSiC and outperforms other deconvolution methods when applied to snRNA-seq data. DeTREM enables accurate cell-type deconvolution in situations where scRNA-seq data are not available. This modification improves characterization cell-type specific effects in brain tissue and identification of cell-type abundance differences under various conditions.

**Supplementary Information:**

The online version contains supplementary material available at 10.1186/s12859-023-05476-w.

## Background

Single-cell RNA-sequencing (scRNA-seq) has proven to be an integral method to study developmental and disease biology in humans and lower organisms [1, 2]. Researchers can characterize rare cell-types, identify novel cellular subtypes, and discover possible relevant interactions between cells by profiling the transcriptome of individual cells. By comparison, bulk tissue RNA-sequencing (bulk RNA-seq) measures the average expression of genes in the tissue sample. Expression differences between bulk RNA-seq samples can be due to RNA expression changes within cells, differences in cellular makeup, sequencing differences, or a combination of these factors. Single-nuclei RNA-sequencing (snRNA-seq) is a newer technique that measures transcriptomes in individual nuclei isolated from cells [3]. While both approaches typically capture RNA with poly-A tails, snRNA-seq does not capture cytoplasmic RNA and thus represents cell state differently. Although targeting nuclear RNA is reasonable for many gene expression analyses, snRNA-seq is particularly useful in tissues for which scRNA-seq data is difficult to obtain (e.g. frozen brain). It can be difficult to disassociate individual cells from brain tissue for scRNA-seq, but individual nuclei can be more easily isolated for snRNA-seq [3]. This technical consideration is reflected in many recent Alzheimer disease (AD) studies that use snRNA-seq rather than scRNA-seq data [4–6]. In addition, snRNA-seq is still much more expensive than bulk RNA-seq [7], therefore current snRNA-seq data generally contain no more than a few dozen samples. On the other hand, bulk RNA-seq data are currently available for as many as several thousand frozen human brain specimens, and this number is rapidly growing. Applying deconvolution methods to estimate cell type proportions from bulk-RNAseq data leverages the relative abundance of bulk RNA-seq data and the cell-type specificity of scRNA-seq.

Computational deconvolution methods leverage the cellular specificity of scRNA-seq to quantify cell-type or cell subtype proportions within bulk RNA-seq data. With a single tissue-specific scRNA-seq reference dataset, bulk RNA-seq data from that tissue can be deconvoluted without the need for additional sequencing [8]. Several deconvolution methods have been developed including ones using a reference matrix of cell marker genes [9], sorted bulk-seq samples [10], scRNA-seq data, and no reference dataset [11, 12].

**Fig. 1:**
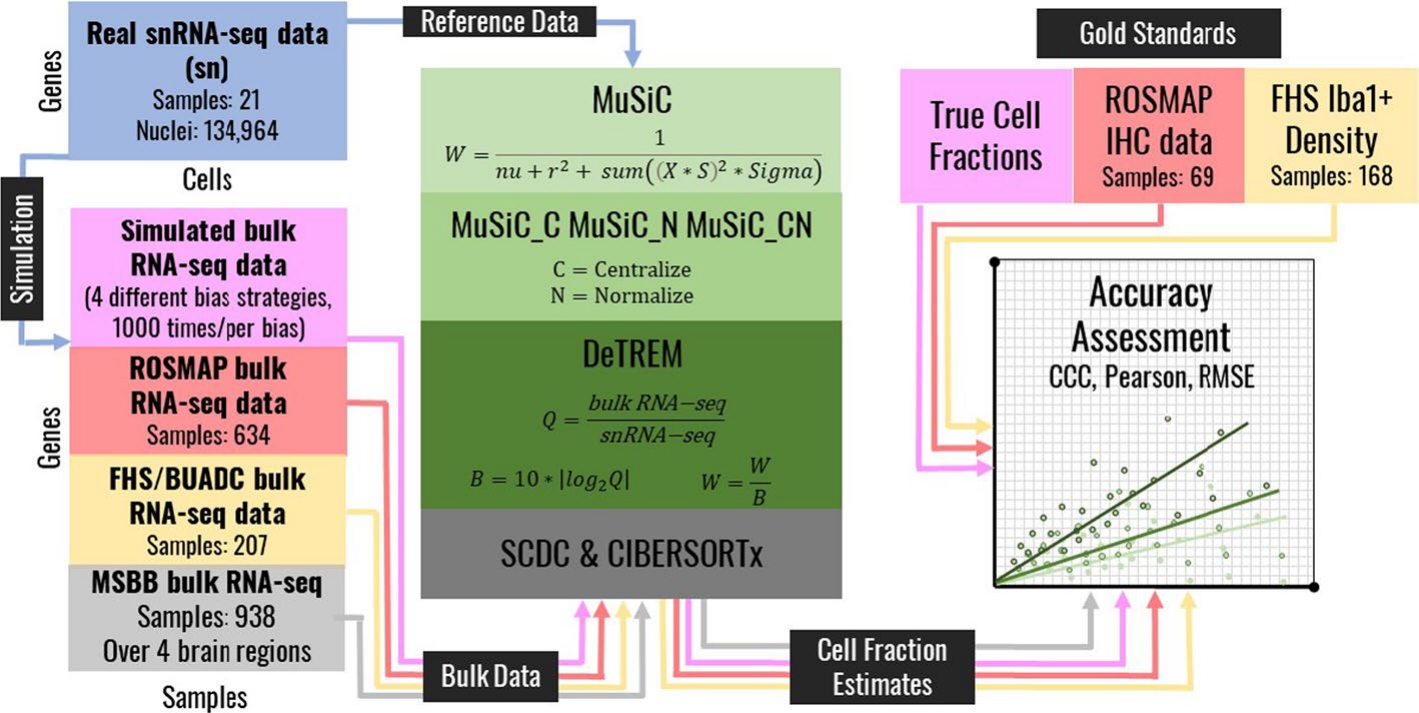
Study design. Bulk RNA-seq data were simulated using real snRNA-seq data. Cell fraction estimates of the simulated and real bulk RNA-seq data were calculated using several MuSiC software variations, SCDC, CIBERSORTx, and a DeTREM. Next, the accuracy of the cell fraction proportions obtained using each deconvolution method for each dataset was determined by comparisons with experimentally derived data as follows: (1) simulated bulk RNA-seq data were compared to true cell fractions, (2) ROSMAP brain data were compared to matched IHC measurements, and (3) FHS/BUADRC brain data were compared to matched Iba1 + density measurements

MUlti-Subject SIngle Cell (MuSiC), one of the widely used deconvolution methods, employs weighted non-negative least squares regression to estimate cell proportion [13]. MuSiC does not require pre-selected cell marker genes, but effectively selects reliable and predictable marker genes through its gene weighing scheme. It performs similarly to other deconvolution methods such as DWLS and SCDC that use scRNA-seq data as a reference [14]. Although MuSiC was designed to deconvolute bulk RNA-seq data using a scRNA-seq reference dataset, it can also be used with a snRNA-seq reference data noting that cell-type proportions are often estimated to be zero perhaps because of sequencing limitations of snRNA-seq or the relatively lower quality of RNA extracted from nuclei compared to whole cells. MuSiC can compensate for the significant expression differences between snRNA-seq and bulk RNA-seq with its optional normalization parameter (MuSiC_N) which divides input expression values by their standard deviations, centralization parameter (MuSiC_C) which subtracts the input expression values by their averages, or a combination of the two (MuSiC_CN). While these parameters increase estimation accuracy for some cell-types or samples, we show that they do not consistently improve estimation accuracy over default MuSiC. To address this problem, we developed software called Deconvolution with Target and Reference differences Extending MuSiC (DeTREM) which incorporates an alternative weight scheme that better compensates for sequencing differences when using snRNA-seq or other disparate reference datasets. DeTREM increases the accuracy of cell-type proportion estimates over MuSiC and performed similarly to two recently developed deconvolution algorithms [15, 16] in simulated data. We further validated the deconvolution accuracy of DeTREM using bulk RNA-seq data, cell density measurements, and immunohistochemistry (IHC) data generated from human brain tissue (Fig. 1).

## Results

### Simulating biased and unbiased bulk RNA-seq data

In order to determine which of our simulated bulk datasets best reflect real bulk RNA-seq brain data, we plotted them with snRNA-seq data for each gene (Additional file 1: Fig. S1). The average expression for each gene in the unbiased simulated bulk data was highly correlated with the average single-nuclei RNA expression (Additional file 1: Fig. S1b), which is dramatically different from the real data (Additional file 1: Fig. S1a). By contrast, simulations 1, 2 and 3, which were biased by gamma distributions (Additional file 1: Fig. S1c-e), and simulation 4, which was biased using FHS bulk-seq expression (Additional file 1: Fig. S1f), show scatter similar to that observed in real bulk data (Additional file 1: Fig. S1a). These results show that the biased simulated data, particularly simulation 2 (Additional file 1: Fig. S1d), are sufficiently similar to actual human brain bulk RNA-seq data for assessing deconvolution performance. The results of simulation 4, with a bias distribution calculated by dividing the normalized average gene expression of snRNA-seq by that derived bulk RNA-seq data, is similar to simulations 1–3.

### DeTREM improves accuracy of deconvoluted simulated data

**Fig. 2:**
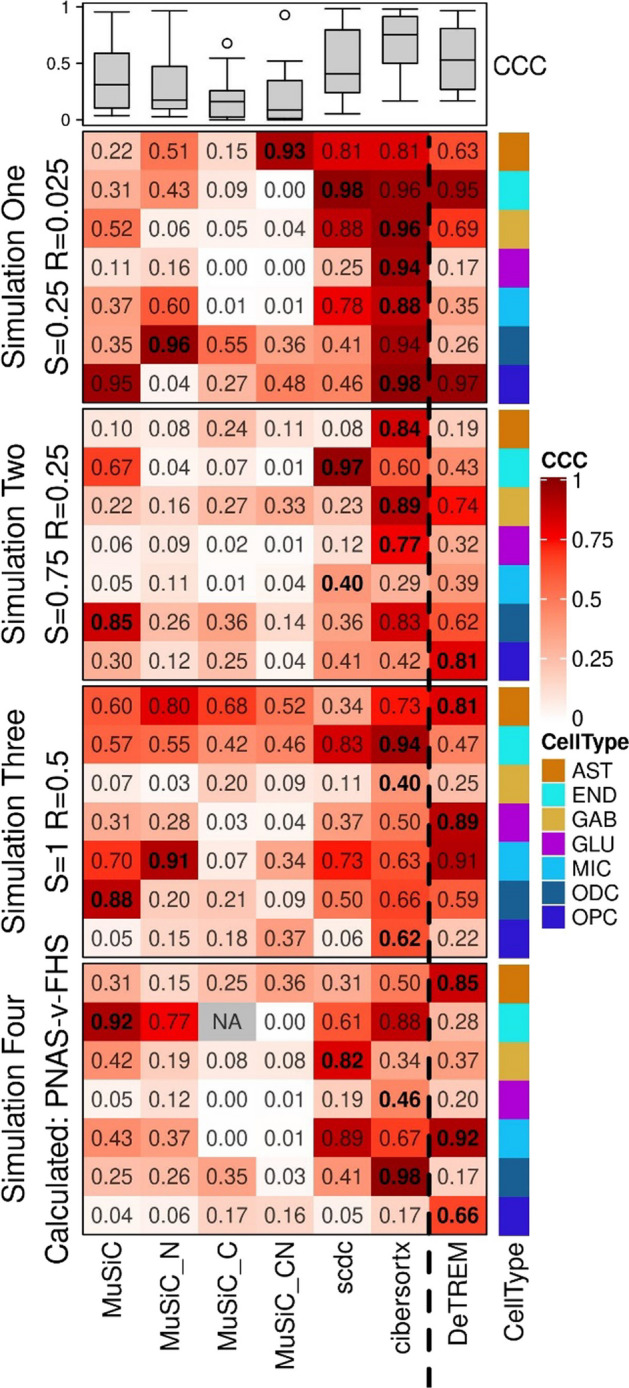
Deconvolution accuracy assessed for four scenarios of simulated data. Heatmap color shows the concordance correlation coefficient (CCC) between true and estimated cell-type percentages. Each column shows the deconvolution method with versions of MuSiC, SCDC, and CIBERSORTx separated from DeTREM by a dashed line. Rows delineate the seven cell-types assayed as indicated according to the color coding in the key. Results for the method with the highest CCC in each condition are bolded. One set of estimates with zero variance is marked as NA. Box plots indicate the aggregated CCC values for each method

Deconvolution of simulated data using DeTREM was more accurate (i.e., greater concordance between true and simulated data) for the majority of the cell-types using MuSiC with default parameter settings compared to the same software with varied parameter settings (MuSiC_C, MuSiC_N, MuSiC_CN) especially for comparisons of GABAergic neurons (GAB), glutamatergic neurons (GLU) and oligodendrocyte precursor (OPC) cell-types (Fig. 2). In contrast, DeTREM, showed increased concordance between simulated and estimated cell-type proportions compared to MuSiC (Two-sided paired t-test *p* = 0.014). In particular, the deconvolution accuracy was higher using DeTREM compared to MuSiC for microglia (MIC), astrocytes (AST), and oligodendrocyte precursors across all four simulations. DeTREM also performed better than MuSiC for both neuron types. For the rarer endothelial cell-type (END), the performance of DeTREM compared to MuSiC was better in simulation 1 (CCC = 0.95 versus 0.31), less well in simulations 2 (CCC = 0.43 versus 0.67) and 4 (CCC = 0.28 versus 0.92), and about the same in simulation 3 (CCC = 0.47 versus 0.57). Although the accuracy of MuSiC with or without variable settings was higher in some simulations or cell-types, DeTREM was more consistently accurate across simulations and different cell-types. This improvement is especially pronounced for both types of neurons tested. Generally, SCDC (mean CCC = 0.48) performed better than MuSiC (mean CCC = 0.38) and worse than DeTREM (mean CCC = 0.54). SCDC performed particularly well estimating endothelial cell-type fraction and outperformed DeTREM in each simulation. CIBERSORTx performed better in all cell types (mean CCC = 0.70) than DeTREM and MuSiC. We performed the same accuracy assessment with Pearson’s r and root mean square error (RMSE) and again find DeTREM’s cell-type abundance estimates more accurate than MuSiC or its variants: average r = 0.96 for DeTREM, 0.88 for MuSiC, 0.95 for SCDC, and 0.96 for CIBERSORTx; average RMSE = 0.061 for DeTREM, 0.096 for MuSiC, 0.079 for SCDC, and 0.033 for CIBERSORTx (Additional file 1: Fig. S2).

### Comparison of cell fractions estimated by deconvolution with cell fractions measured by IHC

**Fig. 3:**
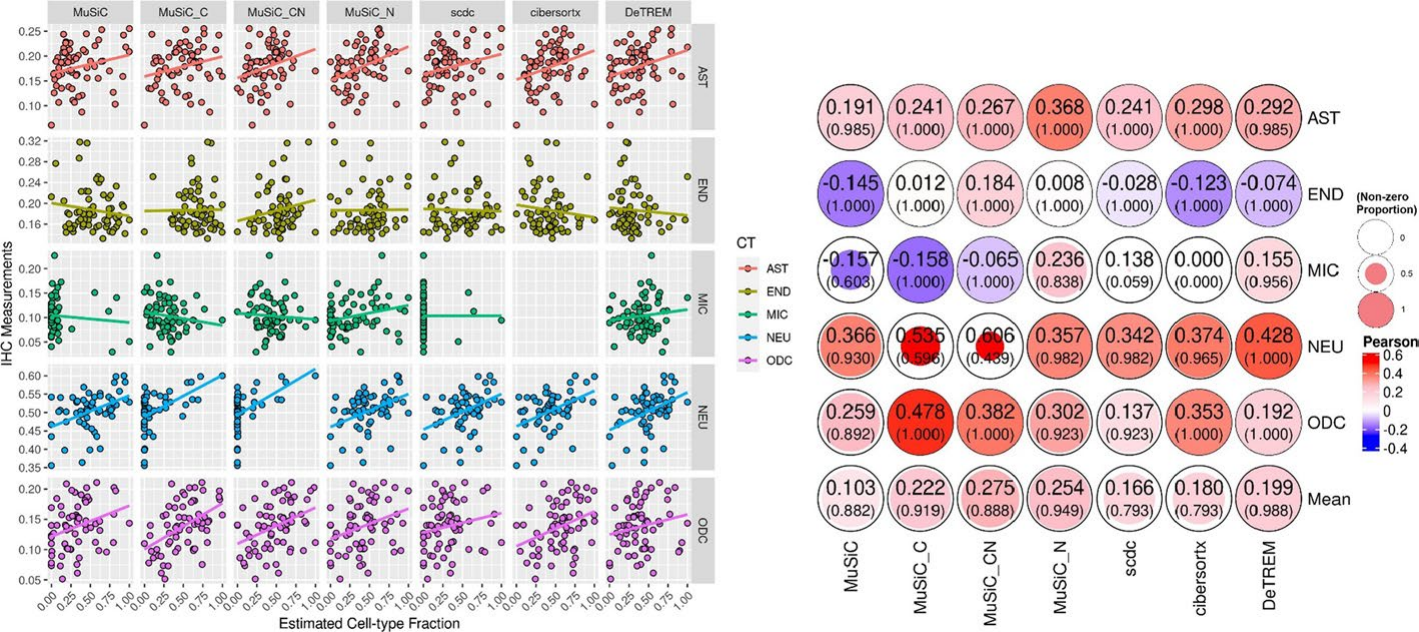
Deconvolution accuracy assessment using 69 matched IHC and ROSMAP bulk RNA-seq samples of the ROSMAP. ell-type fraction estimates from seven bulk RNA-seq deconvolution runs are plotted against cell marker IHC measurements from matched samples in the left panel. Each column shows a different deconvolution method: MuSiC, MuSiC its “C” and “N” parameters, SCDC, CIBERSORTx, and DeTREM. IHC measurements are scaled linearly from zero to one. Neuron (NEU) estimates are obtained by adding glutamatergic and GABAergic neuron percentages. A linear model trend line is shown for each plot. The right panel shows Pearson’s correlations between cell type percentage estimates and IHC measurements for non-zero estimates. The proportion of non-zero estimates are indicated in parentheses. These values were averaged for each method and shown in the bottom row

To assess deconvolution accuracy in human brain data, we compared the cell-type percentages estimated by each method to IHC values in the same bulk samples (Fig. 3). IHC measurements are more highly correlated with DeTREM’s cell type percentage estimates than MuSiC for all cell types except oligodendrocytes. When applied using optional parameters, MuSiC’s estimates correlate more highly with IHC measurements but calls more cell-types as absent than DeTREM. Specifically, DeTREM dramatically reduced the quantity of zeroes called in glutamatergic neurons (1 vs. 69), microglia (18 vs. 271), and oligodendrocytes (ODC) (0 vs. 47) in the ROSMAP bulk dataset tested when compared with MuSiC (Additional file 1: Fig. S3). MuSiC_C and MuSiC_CN have low zero counts for microglia (0 and 2), but a particularly high quantity of zeroes for glutamatergic neurons (321 and 375). Although MuSiC_N’s estimates show the highest correlation with IHC measurements and it called the fewest zeroes of any MuSiC derivative, it called 124 more cell-types as absent than DeTREM in the dataset. SCDC and CIBERSORTx performed similarly to DeTREM in this comparison, however, IHC measurements are more highly correlated with the oligodendrocyte cell type percentage estimated by CIBERSORTx (r = 0.35) than DeTREM (r = 0.19). Also, the zero counts of microglia estimated by SCDC (577) and CIBERSORTx (633) are much higher than those estimated by DeTREM or any MuSiC variant (Additional file 1: Fig. S3).

To validate DeTREM’s lower missingness rate (i.e., zero count estimates) compared to other deconvolution algorithms, we deconvoluted bulk RNA-seq data for four brain regions that were obtained from 300 individuals in the Mount Sinai Brain Bank (MSBB). We observed that DeTREM yielded the smallest percentage of cell-types predicted to have an abundance of zero over all samples in each brain region compared to the other methods (Additional file 1: Fig. S4).

**Fig. 4:**
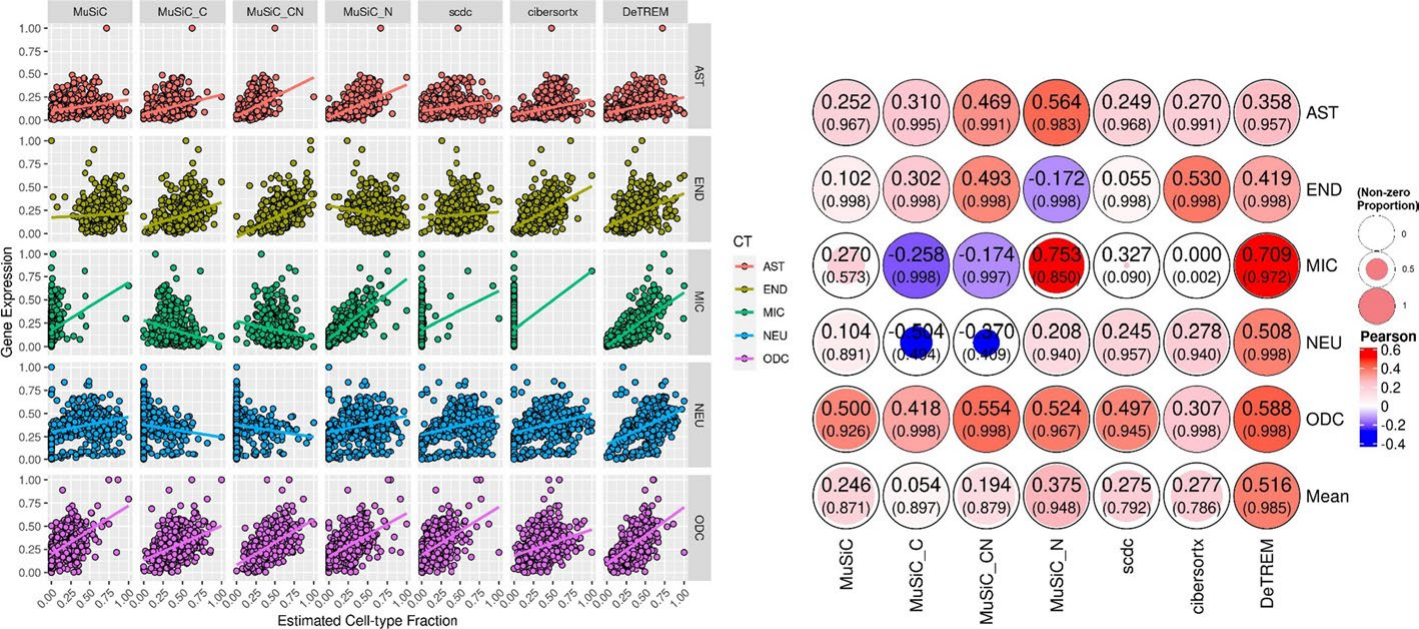
Accuracy assessment with marker gene expression. econvoluted cell-type fractions compared with their marker gene expression from 634 samples of the ROSMAP bulk RNA-seq data. In the left panel, cell-type fraction estimates from seven bulk RNA-seq deconvolution runs are plotted against cell marker expression from the same samples. The cell-type markers are GFAP for astrocytes (AST), PECAM-1 for endothelial cells (END), IBA1 for microglia (MIC), NeuN for neurons (NEU), and Olig2 for oligodendrocytes (ODC). Each column shows a different deconvolution method: MuSiC, MuSiC with its “C” and “N” parameters, SCDC, CIBERSORTx, and DeTREM. Cell-type marker expression is scaled linearly from zero to one. NEU estimates were obtained by summing glutamatergic and GABAergic neuron percentages. A linear model trend line is shown for each plot. The right panel shows Pearson correlations between cell type percentage estimates and marker gene expression for non-zero estimates. The proportion of non-zero estimates are indicated in parentheses. These values were averaged for each method and shown in the bottom row

Comparison of the scaled RNA expression of the cell-type marker genes to the estimated cell fractions of the same samples showed that the average performance across all five cell-types was much higher for DeTREM (average r = 0.52) compared to MuSiC (average r = 0.25), MuSiC_C (r = 0.054) and MuSiC_CN (r = 0.19), MuSiC_N (r = 0.38), SCDC (r = 0.28), and CIBERSORTx (r = 0.28) (Fig. 4). Notably, MuSiC tested with any of the optional parameters yielded a negative correlation for at least one cell-type. DeTREM estimated the fewest absent cell-types in these trials. Additionally, DeTREM performed better in each cell type than SCDC and CIBERSORTx with the exception of endothelial cells using CIBERSORTx. Overall, deconvoluted cell fraction estimates were more strongly correlated with RNA expression of cell marker genes than with cell fractions estimated by IHC. This finding is consistent with the relatively low correlation between protein and mRNA level expression observed in this and other studies [16] (Additional file 1: Fig. S5).

**Fig. 5:**
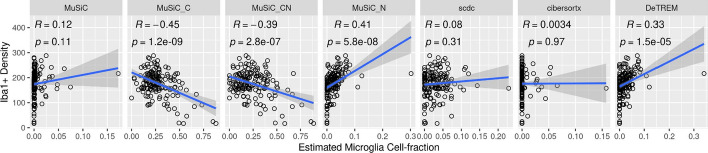
Concordance of microglia density measurements with deconvoluted cell fraction. Correlation of microglia cell fractions estimated by deconvolution with microglia cell density were determined using the Iba1 microglia cell marker in the same individual and region of the FHS/BU-ADRC brains. The trend line was fitted using a linear model and the corresponding 95% confidence interval is indicated with gray shading

Because microglia play an important role in the pathogenesis of multiple neurodegenerative diseases, we compared microglia cell fraction estimates and Iba1 + cell density measurements from matched individuals and regions in the FHS/BUADC bulk RNA-seq dataset (Fig. 5). These variables were uncorrelated using MuSiC (r = 0.12, *p* = 0.11), SCDC (r = 0.08, *p* = 0.31), and CIBERSORTx (r = 0.0034, *p* = 0.97) and significantly inversely correlated using MuSiC_C (r = -0.45, *p* = 1.2 × 10^–9^) or MuSiC_CN (r = -0.39, *p* = 2.8 × 10^–7^). In contrast, estimated microglia cell fraction and cell density were significantly positively correlated using DeTREM (r = 0.33, *p* = 1.5 × 10^–5^) and MuSiC_N (r = 0.41, *p* = 5.8 × 10^–8^).

## Discussion

Cell-type deconvolution or in silico tissue dissection has become a common strategy to estimate cell-type composition in bulk genomics data, particularly bulk RNA-seq data, without the tremendous expense of single-cell or single-nuclei RNA sequencing. Discrimination of cell-types in bulk tissue can improve understanding of the underlying molecular mechanisms of complex diseases and their subgroups. For example, Li et al. deconvoluted bulk RNA-seq data from 18 different types of cancer with CIBERSORTx [16] and identified myofibroblast subtypes as a poor prognostic factor in nine cancer types [17]. A study by Pantano et al. used MuSiC to characterize cell proportion in different stages of non-alcoholic fatty liver disease [18] and found gene expression differences largely explained by cell proportion changes.

The emergence of the MuSiC algorithm and software greatly increased the ability to deconvolute bulk RNA-seq data obtained from a large number of samples. Our modification to MuSiC increases the algorithm’s accuracy when applied using a snRNA-seq reference dataset by decreasing its reliance on genes which we show to be differentially captured between snRNA-seq and bulk RNA-seq. This improvement was evident in both simulated and real human brain bulk RNA-seq data in each cell-type tested, noting that the largest improvement was observed in microglia and neurons. As a result, deconvolution of ROSMAP bulk RNA-seq data yielded fewer cell-types called as absent using DeTREM compared to MuSiC, SCDC, and CIBERSORTx. In addition, we identified a worrying negative correlation between Iba1 + measurements and the microglia cell fraction estimates when MuSiC was applied with its centralization parameter, and deconvolution conducted using MuSiC with its normalization parameter yielded a negative correlation between the endothelial cell fraction estimates and marker gene expression. Enhancements to the algorithm in DeTREM corrected both problems. SCDC and, in particular, CIBERSORTx performed well in our analysis of simulated data and the correlations of cell fraction proportions estimated using these programs with those determined by IHC were similar to those obtained using DeTREM. However, both algorithms predicted that very few samples have microglia and performed worse than DeTREM when correlating estimated cell fraction with marker gene expression.

Findings in this study suggest that DeTREM is better optimized than MuSiC, SCDC, or CIBERSORTx for deconvoluting snRNA-seq data. This conclusion is consistent with a recent study showing that the deconvolution accuracy of human cortex bulk RNA-seq data using a snRNA-seq reference and MuSiC was less than an approach using pre-selected marker gene expression as a proxy for cell fraction [19]. The improved accuracy of DeTREM for deconvolution with snRNA-seq data also incentivizes the use of snRNA-seq rather than scRNA-seq data because some rare cell-types may be better captured and characterized by snRNA-seq [20]. Compared to scRNA sequencing, snRNA sequencing enables analysis of cell-types, states, and functions in challenging tissue sources, for example, archived frozen clinical and post-mortem specimens. In addition, snRNA sequencing has better cellular coverage particularly for complex tissues that cannot be easily dissociated, such as brain. Despite apparent advantages of snRNA-seq compared to scRNA-seq data for biological investigation, discrepancies arising from cell-type deconvolution using each type of RNA-seq data obtained from the same source have not been well characterized, perhaps because of the paucity of data for enough subjects required to make such comparisons. Thrupp et al. found significant capture rate differences for microglia activation genes from analyses of snRNA-seq and scRNA-seq data derived from four individuals [21] suggesting that the accuracy of deconvolution using these two types of RNA-seq data will vary by cell type.

This study has several notable strengths. Our simulation of a large bulk RNA-seq dataset allowed a standardized and robust evaluation of the performance of MuSiC and DeTREM in a variety of realistic scenarios of bias in the RNA-seq data. We demonstrated an improvement in deconvolution accuracy using DeTREM by comparing cell fraction estimates with cell-type proportions measured at a protein level by IHC, noting that other implementations of MuSiC, in particular MuSiC_N, showed improved accuracy compared to the base MuSiC software. Furthermore, we showed that cell proportion estimates based on deconvolution using DeTREM were more accurate to those obtained using cell-type-specific marker gene expression levels than all other versions of MuSiC, as well as SCDC and CIBERSORTx.

Several caveats should also be considered. First, marker gene expression, IHC abundance, and directly measured cell density are not necessarily highly correlated. For example, DeTREM’s endothelial cell estimates were poorly correlated with *PECAM-1* IHC measurements but showed the strongest correlation with *PECAM-1* expression. Similarly, CIBERSORTx’s estimate of oligodendrocyte proportion was highly correlated with Olig2 IHC measurements, but among the algorithms tested was the least correlated with oligodendrocyte marker gene expression. In addition, correlations between estimated cell fractions and both marker gene expression and IHC abundance were greater than those between the IHC and expression measurements. Because these methods do not necessarily measure the same value, even perfectly accurate deconvolution would not reach a correlation or CCC of one. This type of inconsistency highlights the importance of using as many benchmarking methods as possible in deconvolution studies. Second, currently available reference snRNA-seq and scRNA-seq datasets appropriate for this study are relatively small and derived from few subjects and brain regions, however this situation will likely ameliorate in the near future. Finally, this study focused entirely on human brain datasets and thus deconvolution of other tissue types using DeTREM should be investigated further.

## Conclusion

We demonstrated that a modified version of MuSiC, DeTREM, outperforms SCDC, CIBERSORTx, and previous versions of MuSiC for deconvoluting real bulk human brain RNA-seq data. The DeTREM algorithm is ideal for situations where scRNA-seq data are not commonly available, such studies of post-mortem brain tissue. Future studies should examine the performance of DeTREM in other brain regions and tissues, as well as under various sequencing platforms and other conditions.

## Methods

### Overview

To compare the deconvolution performance of MuSiC and two other deconvolution algorithms, SCDC [15] and CIBERSORTx [16], with our modified method (DeTREM), we simulated four bulk RNA-seq datasets of 1,000 samples from a snRNA-seq reference data and introduced bias in different ways. We compared deconvoluted cell fraction estimates from these data with the simulated cell fractions. Next, we compared the deconvoluted cell fraction estimates of bulk RNA-seq samples with immunohistochemical (IHC) measurements of cell abundance from 69 matched samples from the Religious Order Project / Memory and Aging Project (ROSMAP) Study that were obtained from publicly accessible web sites (https://www.synapse.org/#!Synapse:syn4164376 and https://github.com/ellispatrick/CortexCellDeconv). Next, we evaluated the correlation of microglia cell fractions in bulk RNA-seq data with matched Iba1 + cell density measurements derived from 168 participants of the Framingham Heart Study and Boston University Alzheimer Disease Research Center (FHS/BUADRC). We also deconvoluted bulk RNA-seq data for four brain regions obtained from 300 brains in the Mount Sinai Brain Bank (MSBB). The study design is illustrated in Fig. 1.

### Preprocessing human brain snRNA-seq data

Human prefrontal cortex (PFC) snRNA-seq data derived from 12 AD patients and 9 cognitively normal controls created by S. Lau et al. was obtained from GEO (accession number GSE157827) [5]. This dataset has a stronger representation of glial cells with 73,303 non-neuronal nuclei sequenced after filtering compared to an earlier human PFC snRNA-seq dataset comprising 20,205 non-neuronal nuclei [6]. The raw matrix and metadata of the snRNA-seq dataset were analyzed with Seurat toolkit version 3.1.2 [22]. Following the Seurat guided clustering tutorial workflow [23], data were normalized, scaled, and dimensionally reduced using principal component (PC) analysis. Next, data were clustered using the first 10 PCs in a k-nearest neighbor (KNN) approach with a resolution of 0.8. Marker genes for seven cell-types (astrocytes, microglia, oligodendrocytes, oligodendrocyte precursor cells, endothelial cells, and GABAergic and glutamatergic neurons) were linked to these clusters using a heatmap (Additional file 1: Fig. S6a). Clusters expressing markers of a single cell-type were marked and other clusters were re-evaluated by PCA and KNN and re-visualized (Additional file 1: Fig. S6b). Cell-types expressing the markers of a single cell-type were marked and the cells of other clusters were excluded from further analysis.

### Preprocessing human brain bulk RNA-seq data

Bulk RNA-seq data derived from dorsolateral prefrontal cortex (DLPFC) samples collected from 639 participants (266 AD cases, 167 subjects with mild cognitive impairment, and 201 cognitively normal controls) of the Religious Orders Study and the Rush Memory and Aging Project (ROSMAP) were obtained from the Accelerating Medicines Partnership Program for Alzheimer’s Disease (AMP-AD) portal (Synapse ID: syn4164376) [24]. Aligned bam files were converted to fastq files using the FastqTosam function in Picard tools (http://picard.sourceforge.net). Bulk RNA-seq data derived from DLPFC extracted from brains donated by 207 FHS/BUADRC participants (64 autopsy confirmed AD, 129 controls, 14 unidentified) as previously described [25]. Bulk RNA-seq data generated from brain tissue obtained from 300 subjects with a median age of 85 years (79 AD cases, 202 controls, and 19 cognitive status not specified) and characterized by the Mount Sinai Brain Bank (MSBB) were obtained from the AMP-AD portal (Synapse ID: syn3157743) [26]. The data were derived from four brain regions: frontal pole (n = 260, BM10), superior temporal gyrus (n = 239, BM22), parahippocampal gyrus (n = 215, BM36), inferior frontal gyrus (n = 222, BM44). Quality control (QC) of the fastq files from each study was performed using FastQC version 0.11.9 [27] to check over-abundance of adaptors and over-represented sequences. Fastq files that passed QC were aligned to the human reference genome (GRCh38.95) using STAR (version 2.6.1c) which implements 2-pass mapping to increase mapping of splice reads from novel junctions [28]. Gene- and isoform-level measurements were quantified using RSEM version 1.3.1 [29], Bowtie2 version 2.3.4.1 [30], and GRCh38.95 annotation files. RSEM’s expected read count was used for subsequent analyses.

### FHS cortex cell density data generation

Iba1 + cell count in the FHS samples was quantified using histological staining, digital microscopy and analysis with the Aperio ScanScope (Leica) as previously described [31]. Using this system for digital neuropathological quantitation has been validated in previous studies [31, 32].

### Simulated biased and unbiased bulk RNA-seq data

We simulated bulk RNA-seq data with known cell-type percentages to compare the accuracy of each deconvolution method. First, we calculated the average expression ${\mathrm{X}}_{\mathrm{kg}}$ of each gene g for each cell-type k in the snRNA-seq dataset (E1) described above and normalized the total expression for each cell-type (E2). For each simulation, percentages for six of the seven cell-types ${\mathrm{P}}_{\mathrm{k}}$ were randomly selected from uniform distributions with maxima and minima selected to reflect reasonable brain cell-type abundances (E3) (Additional file 1: Table S1).

$$\left(E1\right) {X}_{kg}=\sum_{c\epsilon {C}_{k}}{X}_{cg}\quad \left(E2\right) {X}{\prime}_{kg}=\frac{{X}_{kg}}{\sum_{g=1}^{G}{X}_{kg}} \quad \left(E3\right) {P}_{k} \sim {\varvec{U}}\left({a}_{k},{b}_{k}\right), k=\mathrm{1,2},\dots ,6$$

For the seventh cell-type, GLU neurons, the abundance was set to one minus the sum of other cell-type proportions (E4). These ranges are then multiplied by normalized average expression per gene per cell-type to calculate the relative abundance ${\mathrm{R}}_{\mathrm{g}}$ of each gene g in a simulation (E5) and normalized to the sum of all genes (E6).

$$\left(E4\right) {P}_{7}=1-\sum_{k=1}^{6}{P}_{k}\quad \left(E5\right) {R}_{g}= {P}_{k} {X}{\prime}_{kg}\; \quad \left(E6\right) {R}{\prime}_{g}=\frac{{R}_{g}}{\sum_{g=1}^{G}{R}_{g}}$$

The number of reads for each was selected from a uniform distribution with a minimum of eight million and a maximum of twelve million. Each read is assigned to a single gene by sampling from the relative abundance of each gene ${\mathrm{R}}_{\mathrm{g}}$. This process is repeated for each of 1,000 simulated samples, leading to a simulated bulk RNA-seq dataset with variability from differing cell-type proportions and read sampling.

Because the simulated bulk sequencing dataset represents an unbiased reflection of the snRNA-seq data, we generated bias terms to simulate sequencing differences. Three bias distributions were generated by taking the log2 of one plus a gamma distribution. The gamma distributions have shape parameters of 0.25, 0.75, and 1.0, and rate parameters of 0.025, 0.25, and 0.5 respectively (i.e., simulations 1, 2, 3) (E7).

$$\left(E7\right) {B}_{g}=log2\left(\Gamma \left(a,x\right)+1\right)$$

A fourth bias distribution was generated to represent the difference between bulk RNA-seq and snRNA-seq in real human data (i.e., simulation 4). The overall expression of genes in the single cell$, {\mathrm{X}}_{\mathrm{cg}}$, and bulk$, {\mathrm{Y}}_{\mathrm{jg}}$, datasets are calculated (E8), normalized (E9), and the quotient per gene,${\mathrm{Q}}_{\mathrm{g}}$, is calculated (E10).

$$\left(E8\right) {Y}_{g}= \sum_{j=1}^{J}{Y}_{jg} \ \ \ \ \ {X}_{g}= \sum_{c=1}^{C}{X}_{cg}\quad \left(E9\right) {X}{\prime}_{g}=\frac{{X}_{g}}{\sum_{g=1}^{G}{X}_{g}} \ \ \ \ \ {Y}{\prime}_{g}=\frac{{Y}_{g}}{\sum_{g=1}^{G}{Y}_{g}}$$

These values, which represent the range of differential capture of genes, are used as a bias distribution (E11). In each simulation, bias values for each gene were generated from the corresponding distribution and multiplied by the cell-type signature matrix ${\mathrm{X}}_{\mathrm{kg}}$ before normalization (E12).

$$\left(E10\right) {Q}_{g}= \frac{{Y}{\prime}_{g}}{{X}{\prime}_{g}}\quad \left(E11\right) {B}_{g}={Q}_{g}\quad \left(E12\right){ X}_{kg}= {X}_{kg}{B}_{g}$$

These bias distributions skew the expression of genes to simulate sequencing differences. Expression of approximately equal numbers of genes is increased or decreased by these distributions, but expression of most genes is relatively unchanged (Additional file 1: Fig. S7). In total, we generated five simulated bulk RNA-seq datasets of 1000 samples each, one unbiased, three biased by gamma distribution, and one biased using a quotient between snRNA-seq and bulk RNA-seq.

### Deconvolution procedures

Deconvolution was performed using default settings with MuSiC and our modified method (DeTREM) on the simulated and human brain bulk-seq samples. Additional comparisons were made using MuSiC’s 'centered' (MuSiC_C) and 'normalize' (MuSiC_N) parameters alone and in combination (MuSiC_CN). MuSiC iterates non-negative least squares regression (NNLS), changing weight values for each gene, until the change in precited cell-type proportion falls below an absolute limit or the maximum number of iterations is reached. Each iteration begins with NNLS predicting ${p}_{kj}$, the proportion of cell-type k for subject *j*, given the signature matrix ${\theta }_{kg}$, the average expression of gene g in cell-type *k*, and ${Y}_{jg}$, the average bulk expression of gene g in subject j. It generates residuals per subject and gene ${r}_{jg}$, (E13) and tries to minimize the residual per gene (E14).

$${\left(\mathrm{E}13\right)Y}_{jg}= {r}_{jg}+\sum_{k=1}^{K}{p}_{kj}{\theta }_{kg}\quad \left(\mathrm{E}14\right) {r}_{g}=\sum_{j=1}^{J}{r}_{gj}$$

MuSiC implements a gene weighing schema in order to reduce the impact of poorly matched genes or genes otherwise predicted to be less reliable. It calculates a measure of cross-subject variance per gene in each cell-type, ${\sigma }_{kg}$, and uses it in the weighing schema to reduce the weight of genes that are highly variable between subjects within a cell-type. This reduces the algorithm’s dependence on genes which are demonstrably less reliable as cell-type markers. The value is adjusted by MuSiC’s size factor of each cell type ${S}_{k}$ and signature matrix ${\theta }_{kg}$ to get a measure of cross-subject variability per gene in cell-types in which it is expressed (E15). This focuses the cross-subject variability of a gene on cell-types in which it is most expressed while adjusting for the predicted average expression of each cell-type with ${S}_{k}$.

$$\left(\mathrm{E}15\right) {\sigma }_{g}=\sum_{k=1}^{K}{{(S}_{k}{\theta }_{kg})}^{2}{\sigma }_{kg}\quad \left(\mathrm{E}16\right) {{w}_{g}=\frac{1}{{\sigma }_{g}+{r}_{g}^{2}+\nu }}$$

Finally, the weight for each gene is calculated using ${\sigma }_{g},$ ${r}_{g}$, and a regulation parameter $\nu$, set at 0.0001 (E16). For subsequent iterations the expression values of genes in the original ${Y}_{jg}$ and ${\theta }_{kg}$ are multiplied by the square root of ${w}_{g}$, reducing the importance of genes with high residuals or subject variability in NNLS. DeTREM extends this weighing schema to reduce the importance of genes with high variability between the reference and target datasets.

The quotient between snRNA-seq and bulk RNA-seq capture (E10) was log-transformed to log scale. The absolute value of the result was used as an indicator of the strength of difference between the datasets and multiplied by ten (E17). The objective of these steps is to reduce gene weights that vary between conditions, and any values greater than 1 are reduced to one so that no gene weights are increased (E18). Each iteration’s gene weight is divided by this value (E19) and the iterations are continued. This reduces the weight of genes that vary widely between reference and target datasets while maintaining or only slightly reducing the weights of genes that do not vary considerably.

$$\left(\mathrm{E}17\right) {Q}{\prime}_{g}=10|{\mathrm{log}}_{2}{Q}{\prime}_{g}\ |\ \ \ \ \ \ \left(\mathrm{E}18\right) {Q}{\prime}_{g}=\left\{\begin{array}{c}{Q}{\prime}_{g} \ \ \ if \ \ {Q}{\prime}_{g}<1\\ 1 \ \ \ \ \ \ if \ \ {Q}{\prime}_{g}\ge 1\end{array}\right.\quad \left(\mathrm{E}19\right) {w}{\prime}_{g}= \frac{{w}_{g}}{{Q}{\prime}_{g}}$$

Deconvolution of the same data was also performed using the default settings in SCDC version 0.0.0.9000 [15] and Singularity version 3.8.5–2.el7 of the CIBERSORTx version 1.0 [16] Docker image. Cell fraction estimates from each method and each dataset were aggregated for performance assessment.

Deconvolution performance in each region of the MSBB dataset was assessed by calculating the percent of zero estimates for each cell-type and sample.

### Assessment of DeTREM performance: application to human brain bulk RNA-seq data

The accuracy of the cell fraction estimates of each deconvolution method in each simulation was assessed by calculating the concordance correlation coefficient (CCC) [33], Pearson’s correlation coefficient (r), and root mean square error between true and estimated cell-type percentages. The CCC was the primary metric for assessing simulated comparisons because two measurements of the same value (i.e., cell-type percentages) were compared directly. The Pearson’s r was calculated for comparisons between different measurements of cell-type abundance in the real bulk RNA-seq dataset. Because we assume that each cell-type will be present to some degree in any DLPFC sample, we also quantified the abundance of zero values resulting from each method’s deconvolution of the ROSMAP DLPFC bulk RNA-seq data. Deconvolution accuracy of DeTREM was evaluated by comparing cell fraction estimations derived from brain DLPFC bulk RNA-seq samples [24] with cell abundance measured by immunohistochemistry (IHC) in the same region in 69 matched individuals using publicly available data [19]. The cell-type markers measured by IHC are commonly used as an ad-hoc measure of cell-type abundance in bulk-seq [19].

The IHC dataset included levels of cell-type-specific proteins that mark neurons (NeuN, n = 57), astrocytes (GFAP, n = 65), microglia (Iba1, n = 68), oligodendrocytes (Olig2, n = 65), and endothelial cells (PECAM-1, n = 65). GABAergic and glutamatergic neuron percentage estimates were combined for analysis of NeuN. The IHC proportion estimates were correlated with the equivalent cell-type fraction estimates, scaled from a minimum of 0 to a maximum of 1. Similarly, the correlation was calculated between the scaled cell-type fraction estimates and scaled marker gene expression for each cell-type. Samples for which a cell-type was estimated to be zero were excluded from each correlation determination and separately quantified. We also tested the accuracy of DeTREM using the FHS/BUADC bulk RNA-seq data and Iba1 + cell density measurements from matched samples. Microglia cell fraction was estimated using each deconvolution method, then these estimated cell fractions were correlated with the Iba1 + density measurements from the same individuals and brain regions.

### Supplementary Information

**Additional file 1**. Supplementary table S1 and supplementary figures S1–S7.

## Data Availability

The snRNA-seq reference data produced by S. Lau et al. are available on GEO (accession number GSE157827) [5]. The ROSMAP bulk RNA-seq data are available through the AMP-AD portal (Synapse ID: syn4164376) [24]. ROSMAP IHC measurements of brain cell-types are available on E. Patrick’s GitHub (https://github.com/ellispatrick/CortexCellDeconv) [19]. The MSBB bulk RNA-seq data is available from the AMP-AD portal (Synapse ID: syn3157743) [26]. The Boston University ADRC bulk RNA-seq & Iba1 + measurements are available through the National Institute on Aging Genetics of Alzheimer Disease Data Storage site (https://www.niagads.org/). Framingham Heart Study data are available in dbGaP and/or through application to the FHS Research Committee (https://www.framinghamheartstudy.org/fhs-for-researchers/). The DeTREM package, developed from MuSiC version 0.2.0, is available on GitHub at https://github.com/nkoneill/DeTREM and requires: R (> = 3.3.2), nnls (> = 1.4), and ggplot2 (> = 2.2.1).

## Abbreviations

snRNA-seq: Single nuclei RNA-seq
scRNA-seq: Single cell RNA-seq
PFC: Prefrontal cortex
DLPFC: Dorsolateral prefrontal cortex
CCC: Concordance correlation coefficient
RMSE: Root mean square error
AST: Astrocytes
END: Endothelial cells
GAB: GABAergic neurons
GLU: Glutamatergic neurons
MIC: Microglia
NNLS: Non-negative least squares regression
ODC: Oligodendrocytes
OPC: Oligodendrocyte precursors
