# Supplementary material for: Bulk brain tissue cell-type deconvolution with bias correction for single-nuclei RNA sequencing data using DeTREM
Source: BMC Bioinformatics. 2023 Sep 19;24:349. doi: 10.1186/s12859-023-05476-w (PMC10507917; doi:10.1186/s12859-023-05476-w)

**Additional File 1**

**Table S1**. Cell-type minimum and maximum proportions applied to simulated data. For each simulation each cell-type’s percentage is randomly selected from this range. Glutamatergic neuron abundance is set to bring the total cell-type abundance per simulated sample to one.

| Cell-type | Minimum | Maximum |
| --- | --- | --- |
| Gabaergic Neurons (Inhibitory) | 0.1 | 0.175 |
| Microglia | 0.09 | 0.1 |
| Oligodendrocyte Precursors | 0.1 | 0.12 |
| Astrocytes | 0.1 | 0.15 |
| Endothelial Cells | 0.075 | 0.095 |
| Oligodendrocytes | 0.15 | 0.25 |
| Glutamatergic Neurons (Excitatory) | 0.11 | 0.385 |

**Figure S1**. Validation of simulated data using the average expression of each gene in each dataset. Each gene’s mean normalized expression over all cells in the single-nuclei RNA-Seq dataset (y axis) is plotted against the mean normalized expression for bulk (A) or simulated samples (B-F). Pearson correlation values are shown. (B) Unbiased simulation. (C-E) gamma distributions with shape parameters of 0.25, 0.75, and 1.0 and rate parameters of 0.025, 0.25, and 0.5 respectively. (F) Simulated data with a bias distribution calculated from the FHS bulk RNA-Seq samples.

| A ρ=0.197  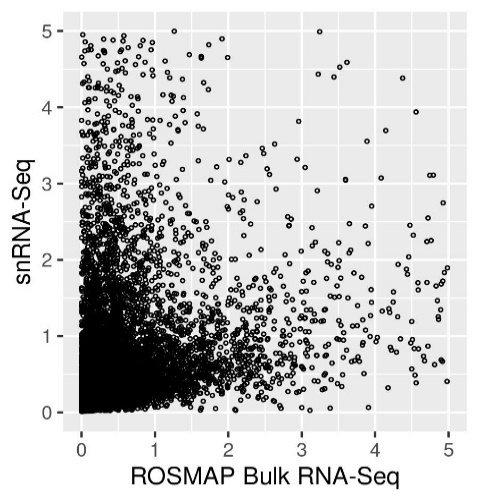 | B ρ=0.995  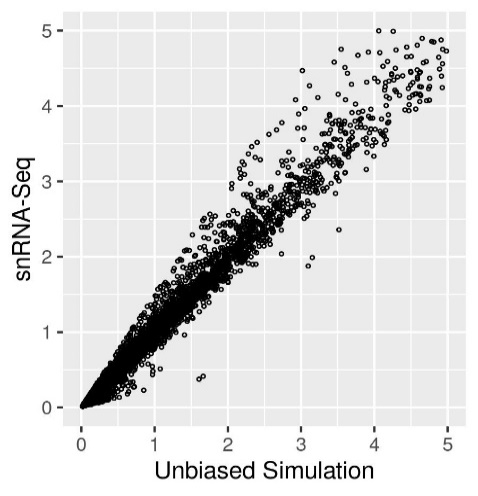 | C ρ=0.482  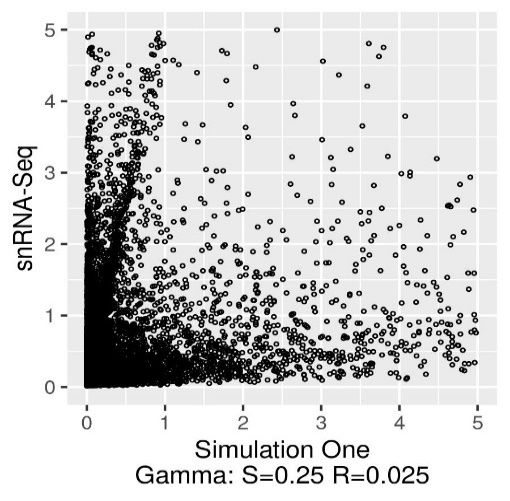 |
| --- | --- | --- |
| D ρ=0.543  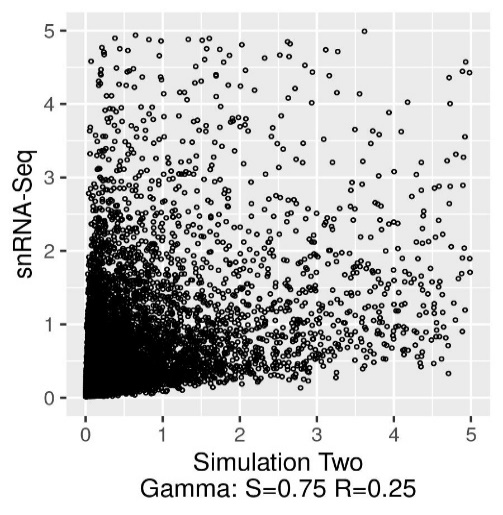 | E ρ=0.575  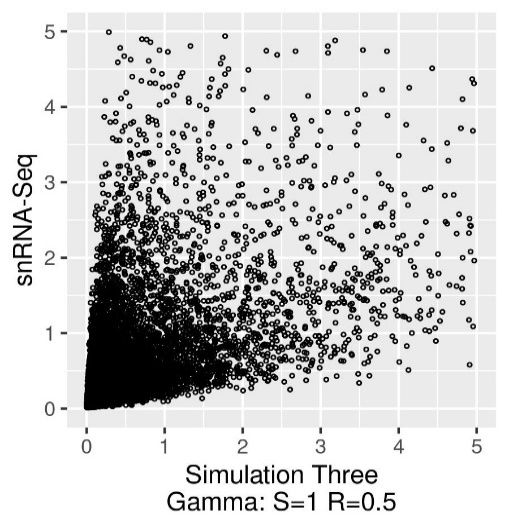 | F ρ=0.318  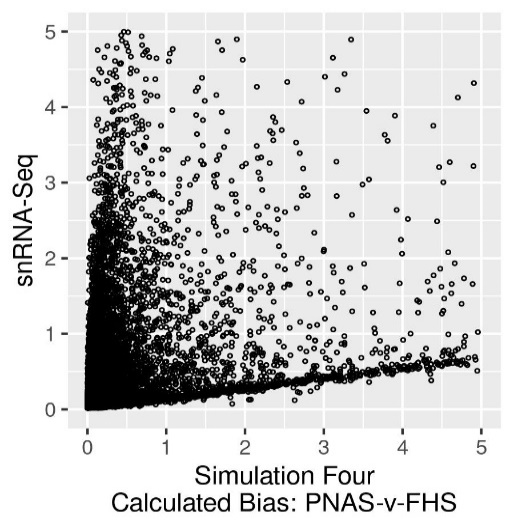 |

**Figure S2**. Deconvolution accuracy assessed for four scenarios of simulated data. Heatmap color shows the Pearson correlation (r) in panel A and root mean square error (RSME) in panel B between true and estimated cell-type percentages. Each column shows a different deconvolution method: MuSiC, MuSiC with centralization “C” and normalization “N”, SCDC, CIBERSORTx, and DeTREM separated by a dashed line. Rows delineate the seven cell-types assayed as indicated according to the color coding in the key. Results for the method with the highest value in each condition are bolded. One set of estimates with zero variance is marked as NA. Box plots indicate the aggregated quality metric values for each method.


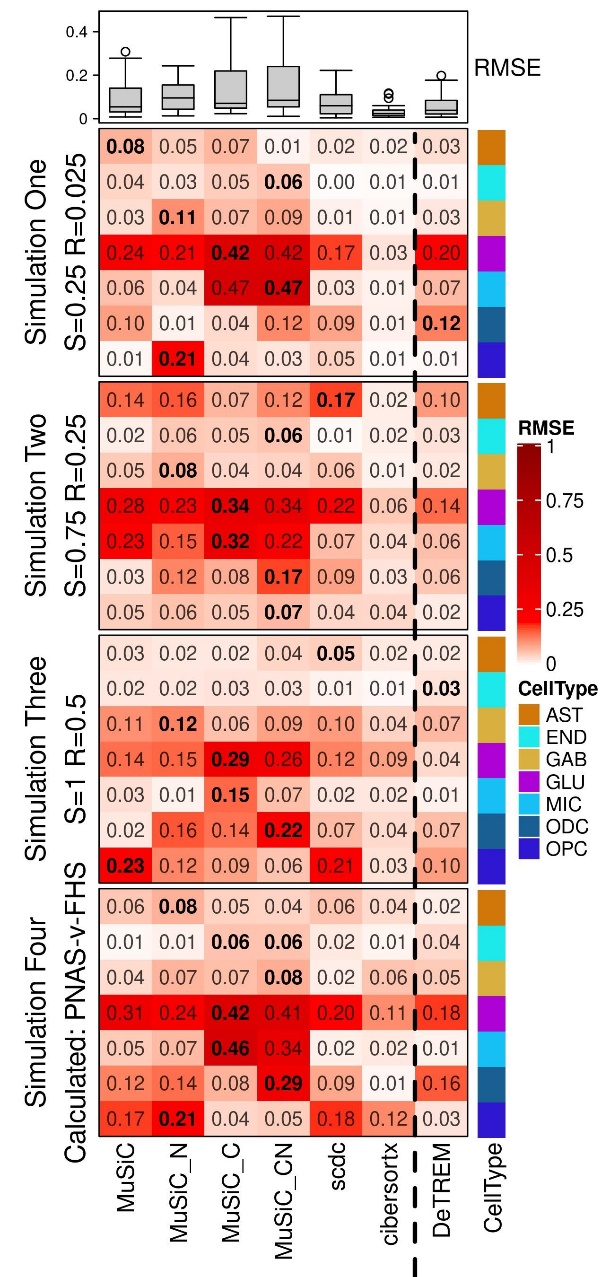

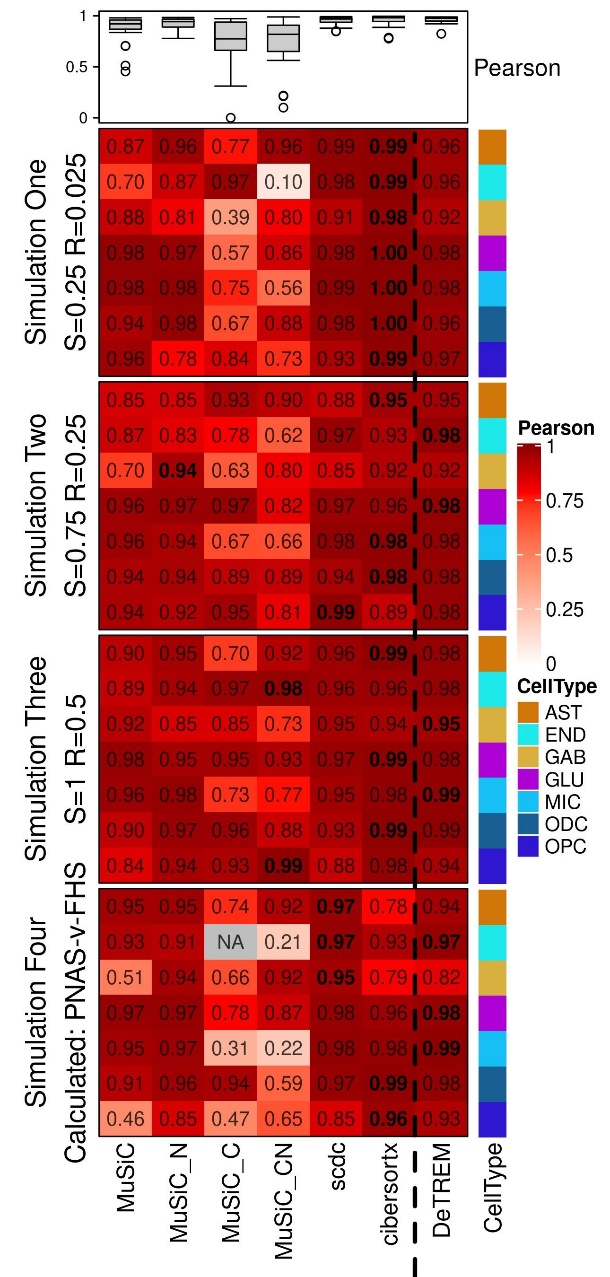


A B

**Figure S3**. Quantity of zeroes in the cell fraction estimates of the ROSMAP bulk RNA-seq dataset for each of seven cell types (AST=astrocytes, END= endothelial cells, GLU= excitatory neurons, GAB= inhibitory neurons, MIC=microglia, ODC=oligodendrocytes, OPC=oligodendrocyte precursors). Zero abundance is shown for each deconvolution method: MuSiC, MuSiC with its “C” and “N” parameters, SCDC, CIBERSORTx, and DeTREM.


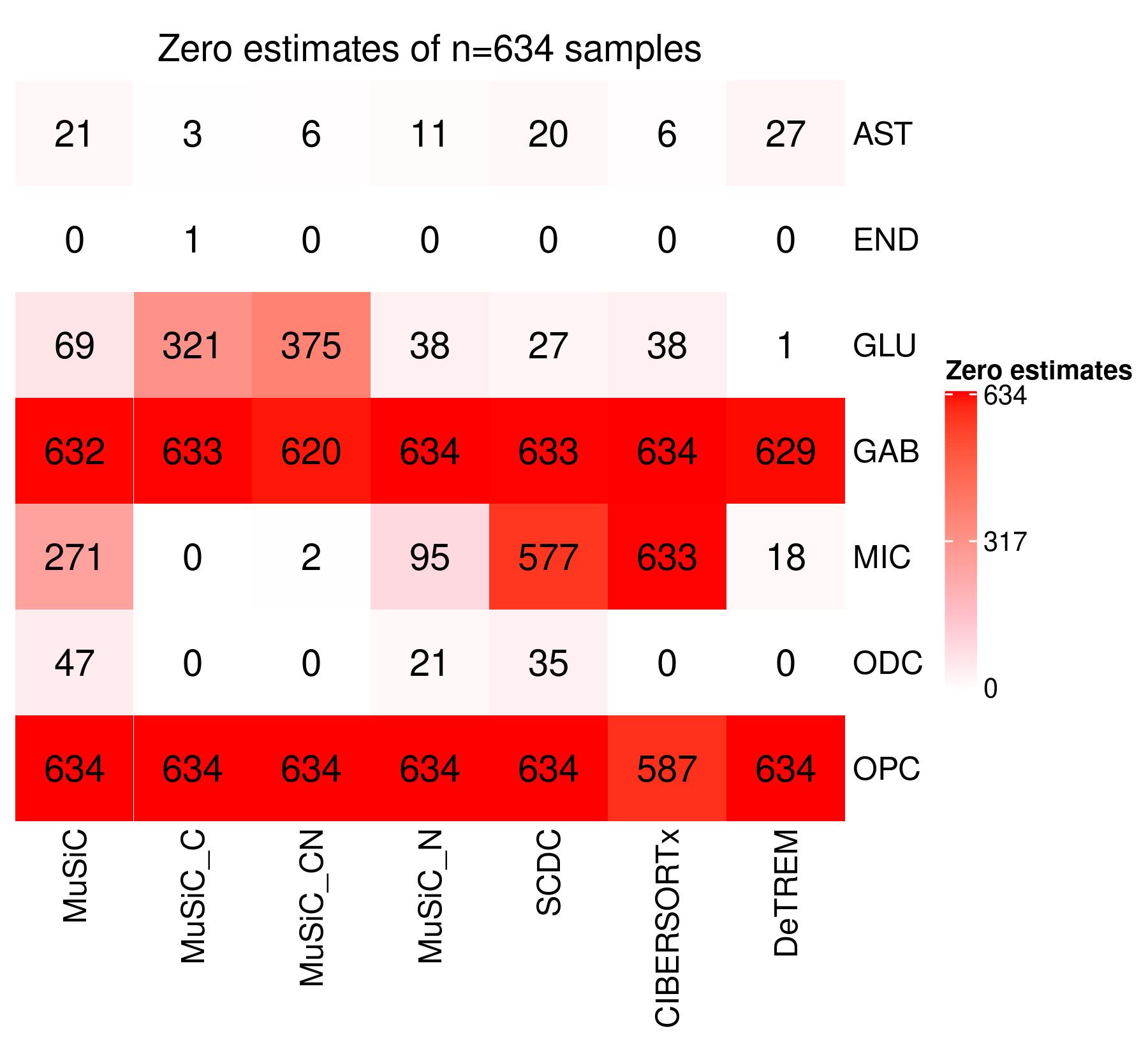


**Figure S4.** The abundance of zero estimates for cell-types among subjects (missingness) for each brain region in the MSBB dataset and each deconvolution method tested. The regions are frontal pole (BM10), superior temporal gyrus (bm22), parahippocampal gyrus (bm36), and inferior frontal gyrus (bm44).


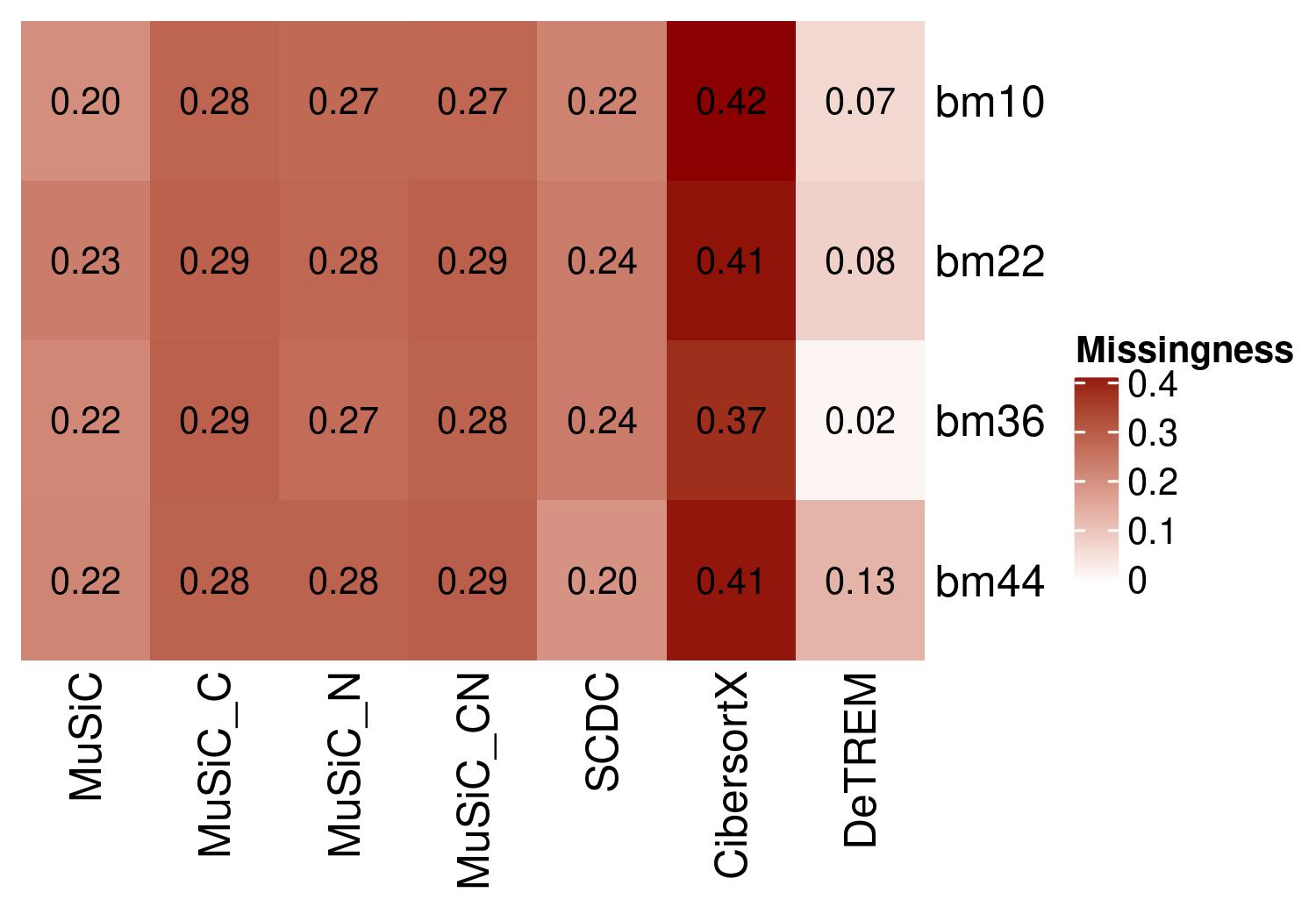


**Figure S5.** Correlation of immunohistochemistry (IHC) protein measurements with RNA-Seq expression of the same genes in 69 matched samples. Values for both IHC measurements and RNA expression are scaled from 0 to 1. Concordance correlation coefficient (CCC) values for each comparison are shown above the plot with an overall CCC = 0.15. NeuN = neurons, GFAP = astrocytes, IBA1 = microglia, Olig2 = oligodendrocytes, PECAM-1 = endothelial cells.


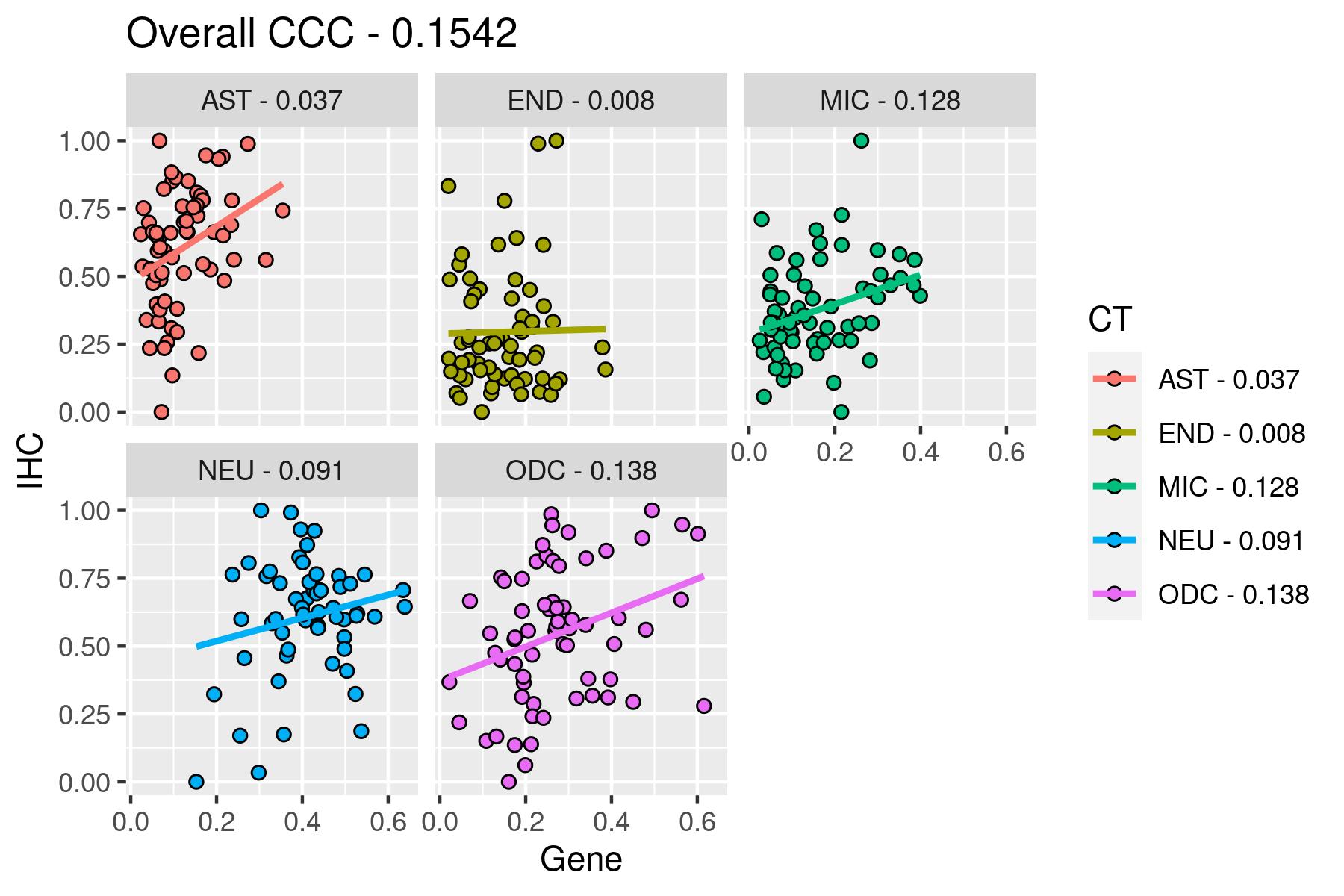


**Figure S6**. Identification of cell types for each cluster. Scaled expression of marker genes for each of seven cell types in the snRNA-Seq data. Rows represent a cell and each group of rows indicated by a common color shown on the left is a cluster. Results for each cell type marker gene are shown in each column. Clusters are identified by cell type according to the key on the right legend or specified as ‘unknown’ in black or ‘mixed’ in white. Cells from ‘unknown’ clusters in panel A were isolated, re-clustered, and cell-typed in B.

1. B.


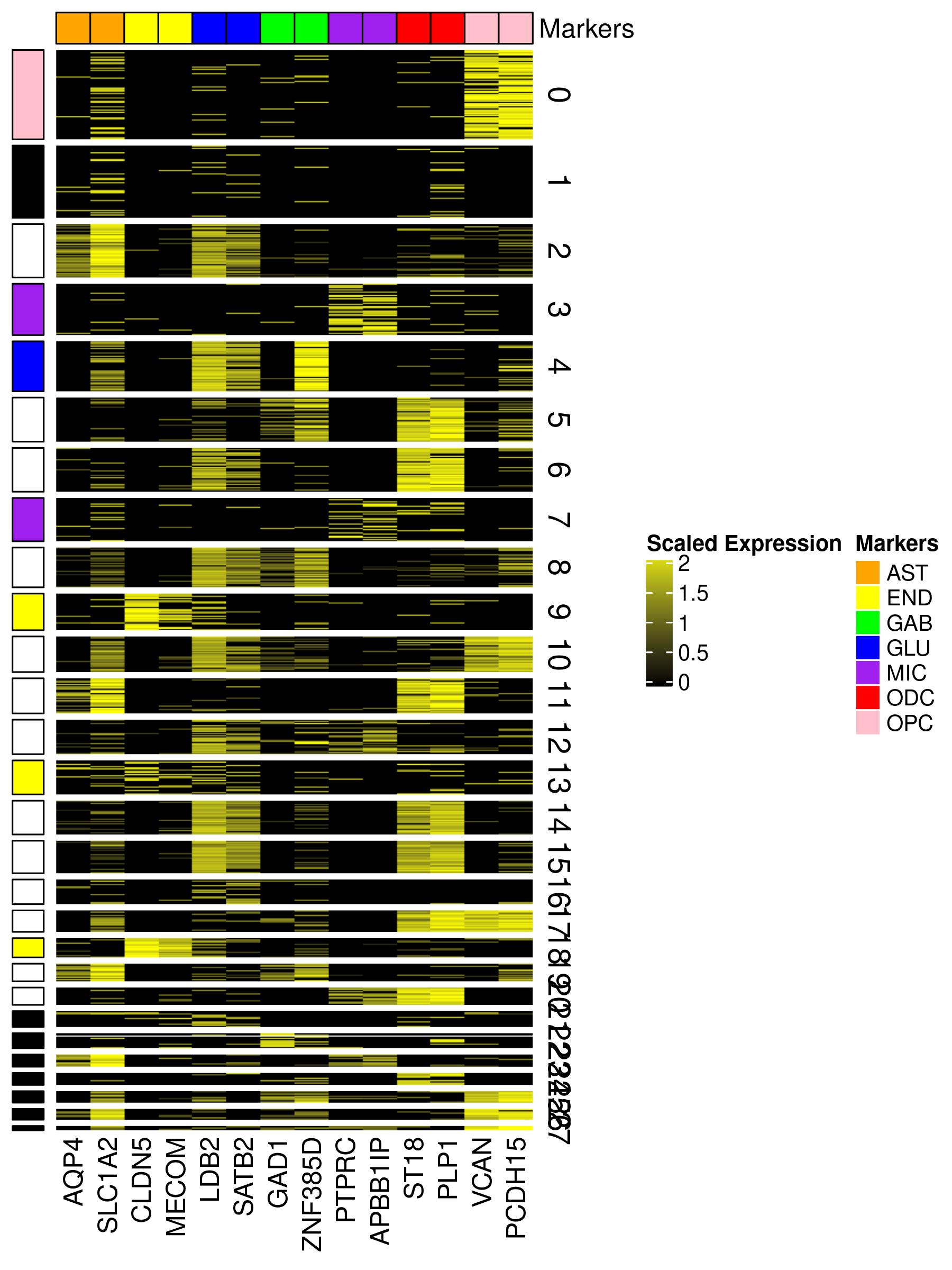

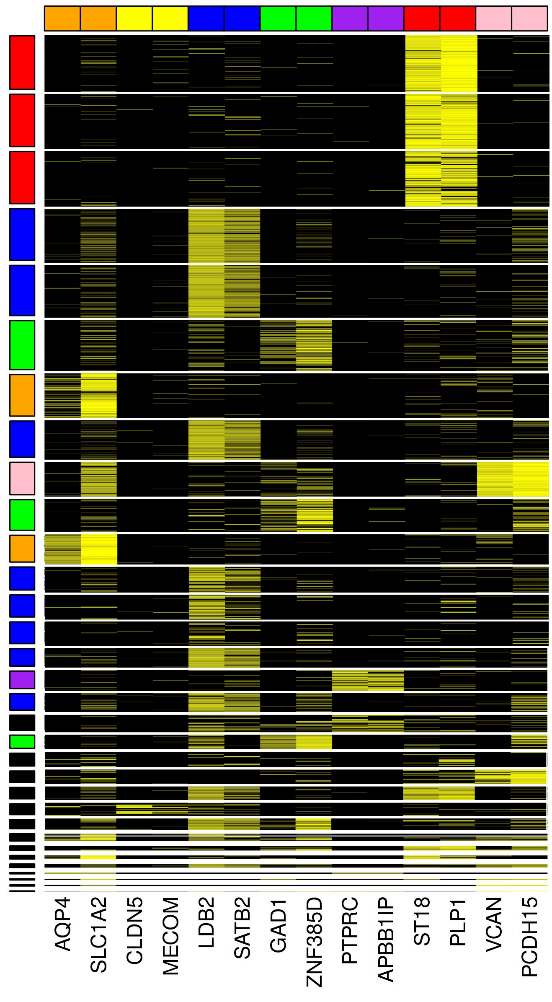


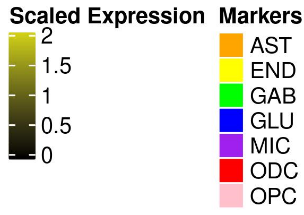


**Figure S7.** Bias distributions used for each simulation of bulk RNA-seq data. Simulations 1-3 are one plus gamma distributions with shape parameters of 0.25, 0.75, and 1.0 and rate parameters of 0.025, 0.25, and 0.5 respectively. A density plot of the log2 of each value is shown.


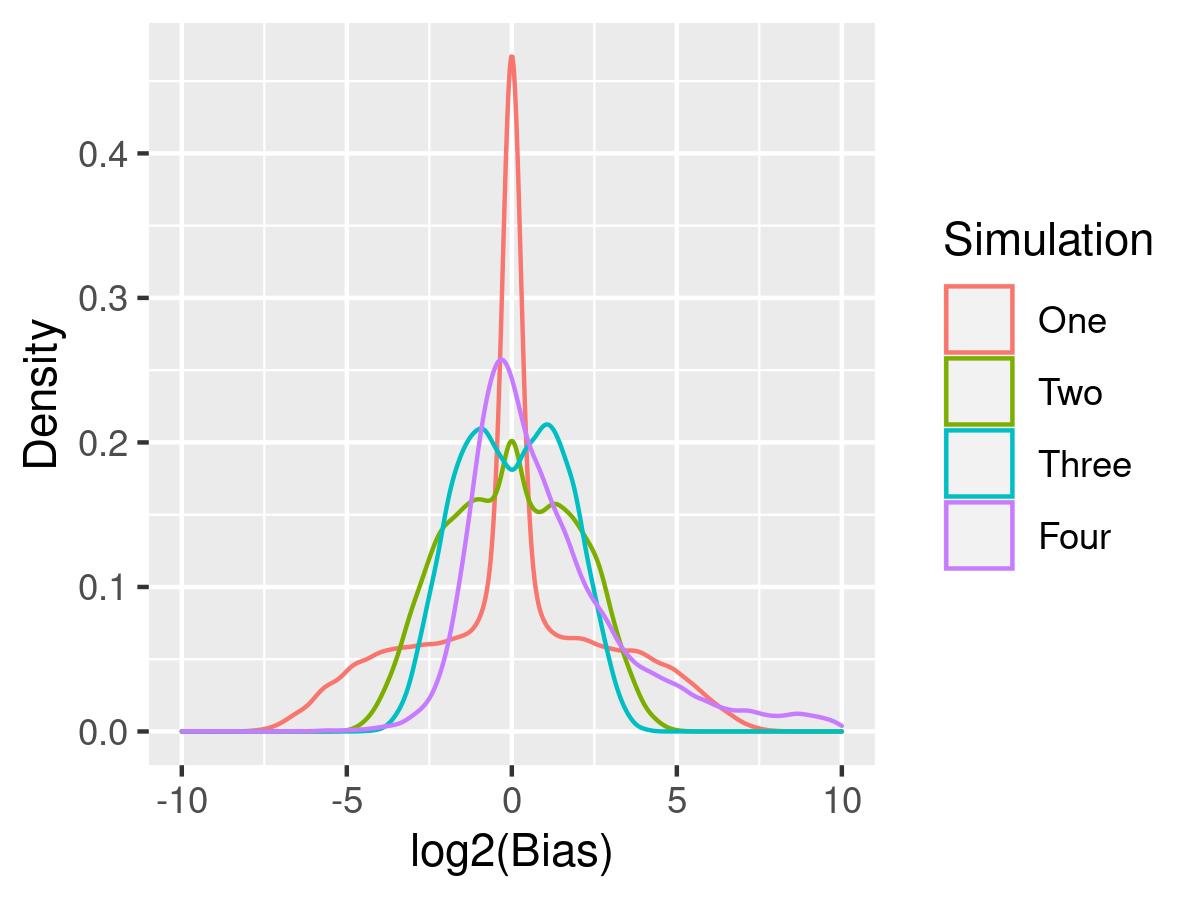

Supplement: Supplementary file 1 — Additional file 1. Supplementary table S1 and supplementary figures S1–S7. [file 12859_2023_5476_MOESM1_ESM.docx]
